# Neonatal-onset familial hemophagocytic lymphohistiocytosis: a case report with genetic confirmation of PRF1 mutations

**DOI:** 10.3389/fgene.2025.1690283

**Published:** 2025-12-16

**Authors:** Xiaoyun Lyu, Pu Wei, Libo Zhu, Wei Zhang

**Affiliations:** Department of Obstetrics, Hangzhou First People’s Hospital of China, Westlake University, Hangzhou, China

**Keywords:** familial hemophagocytic lymphohistiocytosis (FHL), PRF1 gene, neonatal rare disease, multi-organ failure, autosomal recessive disorder, neonatal sepsis

## Abstract

Familial hemophagocytic lymphohistiocytosis (FHL) is a rare, inherited immune-dysregulation syndrome that can present in the neonatal period and progress rapidly without timely recognition. We report a full-term female who developed abdominal distension, fever, hepatosplenomegaly, and coagulopathy within hours of birth, with multiorgan failure leading to death on day 5 despite intensive care and broad antimicrobial coverage. This timing, essentially at birth with death on day 5, is exceptionally rare in FHL2 and clinically instructive for sepsis-like neonatal presentations. Postmortem whole-exome sequencing identified compound heterozygous PRF1 variants, c.1131C>A (p.Cys377Ter) and c.65delC (p.Pro22Argfs*29), confirming FHL type 2. The presentation closely mimicked culture-negative neonatal sepsis, underscoring the diagnostic challenge of distinguishing primary HLH from infectious and metabolic conditions in early life. This case highlights the need for early clinical suspicion and rapid genomic testing in neonates with fulminant, sepsis-like inflammation. While HLH-directed therapy followed by hematopoietic stem-cell transplantation remains the standard pathway to improve survival, the fulminant neonatal course can outpace therapeutic windows, emphasizing the value of streamlined access to urgent genetics and specialist input.

## 1 Introduction

Familial hemophagocytic lymphohistiocytosis (FHL) is a rare autosomal recessive disorder of immune dysregulation characterized by excessive activation of cytotoxic T lymphocytes and natural killer (NK) cells, leading to uncontrolled systemic inflammation, multiorgan dysfunction, and often death if left untreated. The disease is caused by biallelic mutations in genes responsible for the cytotoxic function of immune cells, most commonly *PRF1*, *UNC13D*, *STX11*, and *STXBP2*, which impair the ability of cytotoxic granules to induce apoptosis in the target cells. This results in a persistent cytokine storm and hemophagocytosis, which is a hallmark of the disorder.

Although FHL can present at any point in childhood, the neonatal-onset form is particularly aggressive and often misdiagnosed as neonatal sepsis due to overlapping clinical features such as fever, hepatosplenomegaly, cytopenias, and coagulopathy. This resemblance frequently leads to delays in definitive diagnosis and targeted treatment. When sepsis evaluation is negative in a neonate with multisystem involvement, alternative considerations include monogenic autoinflammatory disease (e.g., NLRP3/NOMID, NLRC4), primary immunodeficiency, inborn errors of metabolism (Poretti et al., 2013; Schrier Vergano, 2022), and congenital leukemia (Brown et al., 2019; Brown, 2021). The HLH-2004 criteria remain the standard for clinical diagnosis, although definitive identification requires the molecular confirmation of pathogenic variants in HLH-associated genes.

We report a case of neonatal-onset FHL in a full-term female infant who presented with fulminant symptoms shortly after birth and died despite aggressive medical intervention. Whole-exome sequencing revealed compound heterozygous mutations in *PRF1*, confirming the diagnosis of FHL type 2. This case highlights the diagnostic complexity of HLH in neonates and emphasizes the critical importance of early genetic testing in cases of unexplained systemic inflammation and multi-organ dysfunction in this age group.

## 2 Case presentation

### 2.1 Maternal and perinatal history

A 35-year-old gravida 2, para 0 woman presented with an unremarkable antenatal course aside from advanced maternal age. Her first pregnancy ended in an induced abortion at <10 weeks’ gestation; products of conception were not available for pathologic or genetic evaluation. There is no family history of hemophagocytic lymphohistiocytosis, early neonatal deaths, or known consanguinity. She underwent amniocentesis with chromosomal and SNP microarray analysis, both of which returned normal results. At 35 weeks of gestation, vaginal swabs tested positive for group B *Streptococcus* (GBS). At 40 weeks and 1 day, she was admitted for suspected fetal distress. An oxytocin challenge test (OCT) was performed, yielding negative results; however, premature rupture of membranes occurred during the test. Due to a known penicillin allergy, she received intravenous cefazolin for GBS prophylaxis.

Labor was induced with oxytocin at 9:30 a.m. on 5 May 2025, and active labor commenced at 10:20 a.m. The mother developed low-grade fever (37.6 °C–37.8 °C) during labor and was administered epidural analgesia. Due to cephalopelvic disproportion and persistent occiput posterior positioning, cesarean section was performed on May 6. A female neonate was delivered, weighing 3,480 g, and had Apgar scores of 9, 10, and 10 at 1, 5, and 10 min, respectively. The immediate delivery-room examination was unremarkable without hepatosplenomegaly.

### 2.2 Early postnatal course

Approximately 3 h after birth, the neonate developed abdominal distension and fever (38 °C), prompting transfer to the neonatal intensive care unit (NICU). At that time, the examination documented marked abdominal distension with palpable hepatosplenomegaly, in contrast to the normal findings of the delivery-room examination. Care in the NICU was led by senior neonatologists from the Department of Neonatology. From NICU admission until death, the infant remained under the direct care of the NICU team; neonatology supervised all daily management and coordinated all subspecialty consultations and procedures. The team supervised respiratory management (including endotracheal intubation and invasive mechanical ventilation), hemodynamic stabilization with vasoactive support, bedside echocardiographic monitoring, serial laboratory evaluation, and multidisciplinary discussions regarding escalation of support (including ECMO). Clinical details used in this report were abstracted from the NICU record with departmental permission.

Given the very short diagnostic window and severe coagulopathy, several HLH studies and ante-mortem sequencing were not feasible; see Genetic Analysis (Section 2.6) for details.

### 2.3 Physical examination

Upon admission to the NICU, the infant appeared lethargic and hypotonic. The abdominal examination revealed distension with hepatosplenomegaly, consistent with the evolution noted at ∼3 h of life. The delivery-room abdominal examination was normal; the subsequent documentation of hepatosplenomegaly at ∼3 h most likely reflects the well-recognized limitations of immediate post-delivery palpation and normal neonatal physiology. In term neonates, a palpable liver edge below the right costal margin can be physiologic, and clinical palpation/percussion has limited accuracy for detecting splenic enlargement compared with sonography (Ashkenazi et al., 1984; Wolf and Lavine, 2000; Cessford et al., 2018). Splenomegaly is also an early feature of HLH (Henter et al., 2007). Given the fulminant deterioration and the need for continuous resuscitative care, no dedicated abdominal ultrasound could be performed; had sonography been available, it would have provided greater diagnostic accuracy for splenomegaly than physical examination alone (Cessford et al., 2018). Vital signs were as follows: heart rate, 147 beats/min; respiratory rate, 54 breaths/min; blood pressure, 64/36 mmHg; and SpO_2_ 95% (room air). The anterior fontanelle was flat, with normal tension. The skin showed widespread petechiae and ecchymoses, particularly on the upper limbs, as well as generalized pallor. Respiratory assessment revealed tachypnea, coarse breath sounds, and no signs of retraction, wheezing, or crackling. The cardiovascular examination revealed normal heart sounds without murmurs and a capillary refill time of 3 s. Abdominal examination revealed marked distension, hepatomegaly (3 cm below the right costal margin), splenomegaly (2.5 cm below the left costal margin), and absence of bowel sounds. Neurologically, the neonate exhibited normal tone but had reduced primitive reflexes. A right-sided accessory tragus was noted.

### 2.4 Laboratory investigations

Key investigations with reference values are summarized in Table 1; tests not available prior to death are indicated as NA. These values were consistent with disseminated intravascular coagulation (DIC), myocardial damage, and hepatic dysfunction. Additional HLH-related studies were selectively prioritized; where testing could not be completed, the reasons are specified below. Coagulation studies were markedly deranged (PT/aPTT/TT prolonged) with fibrinogen 0.77 g/L and D-dimer 26,074 μg/L. Inflammatory markers included CRP 5.1 mg/L (institutional neonatal reference value: ≤8 mg/L). This isolated value should be interpreted in light of neonatal CRP kinetics: concentrations typically begin to rise 6–12 h after the inciting stimulus and peak by ∼24–48 h; therefore, early samples may remain within the reference range despite evolving hyperinflammation (Boscarino et al., 2023; Fleiss et al., 2023; Kilpatrick et al., 2025). In addition, preanalytical factors such as hemolysis, lipemia, or icterus can interfere with immunochemical assays; no interference flag was reported for this sample, but we acknowledge this general limitation (Krasowski, 2019). Triglycerides 3.78 mmol/L and serum ferritin >40,000 μg/L met HLH-2004 supportive thresholds (hypertriglyceridemia ≥3.0 mmol/L, hyperferritinemia ≥500 μg/L); fibrinogen 0.77 g/L met hypofibrinogenemia ≤1.5 g/L. sIL-2R (soluble CD25) and NK-cell cytotoxicity, HLH-2004 supportive tests, were not obtained because the infant had life-threatening coagulopathy with ongoing transfusion support; during continuous resuscitation, we minimized non-essential blood draws (Henter et al., 2007). Targeted metabolic screening (TMS/GC-MS) was completed and was negative.

**TABLE 1:** Laboratory investigations with neonatal reference values and HLH-2004 relevance.

No.	Test (domain)	Patient result (units)	Reference values (neonate)	HLH-2004 relevance	Interpretation
1	Hemoglobin (hematology)	132 g/L	134–199 g/L (0–1 month)	—	Mildly low
2	Platelets (hematology)	30 × 10^9^/L	150–450 ×10^9^/L	Cytopenia	Severe thrombocytopenia
3	WBC (hematology)	16.4 × 10^9^/L	9.0–30.0 ×10^9^/L (0–1 month)	—	Within neonatal range
4	PT/aPTT/TT (coagulation)	23.6 s/96.3 s/45.2 s	PT 12.7–26.6 s; aPTT 48.7–134.3 s; TT 14–21 s	Supportive	Coagulopathy/DIC pattern
5	Fibrinogen (coagulation)	0.77 g/L	1.5–4.0 g/L	HLH criterion	Hypofibrinogenemia ≤1.5 g/L
6	D-dimer (coagulation)	26,074 μg/L (FEU)	250–2,810 μg/L (FEU) (0–28 days)	Supportive	Markedly elevated
7	CRP (inflammation)	5.1 mg/L	≤ 8 mg/L	—	Shows reference value as requested
8	Triglycerides (metabolic)	3.78 mmol/L	0.3–1.7 mmol/L	HLH criterion	Meets ≥3.0 mmol/L
9	Glucose (metabolic)	2.3 mmol/L	2.6–5.5 mmol/L	—	Low
10	Lactate (metabolic)	9.7 mmol/L	0.5–2.0 mmol/L	Supportive	Severe lactic acidosis
11	ABG (respiratory/metabolic)	pH 7.23; PaO_2_ 69 mmHg; PaCO_2_ 28.3 mmHg; HCO_3_^−^ 11.5 mmol/L; BE -14.5 mmol/L	pH 7.33–7.49; PaO_2_ 60–80 mmHg; PaCO_2_ 35–48 mmHg; HCO_3_^−^ 18–22 mmol/L; BE -7 to −1	—	Metabolic acidosis with respiratory component
12	Ferritin (HLH supportive)	> 40,000 μg/L	≥25–200 μg/L (lab-dependent)	HLH criterion	Meets ≥500 μg/L threshold
13	sIL-2R (HLH supportive)	NA	—	—	Not obtained before death
14	NK-cell activity (HLH supportive)	NA	—	—	Not obtained before death
15	Blood/CSF cultures (microbiology)	Negative	—	—	—
16	Plasma mNGS (microbiology)	Negative	—	—	—
17	TMS/GC-MS (metabolic screening)	Negative	—	—	No inborn error signal

NA, not available before death; FEU, fibrinogen-equivalent units.

### 2.5 Clinical deterioration and imaging findings

Six hours after NICU admission, bedside echocardiography revealed the following findings:

- Left ventricular dysfunction (EF: 0.47)
- Enlarged left atrium and ventricle
- Patent ductus arteriosus with bidirectional shunt, predominantly right-to-left
- Pulmonary hypertension, with pulmonary artery systolic pressure approximated as systolic BP +6 mmHg

By 9 h post-admission, the infant’s oxygen saturation had dropped to 80%, requiring endotracheal intubation and initiation of PC-SIMV mechanical ventilation. Chest radiography revealed bilateral pulmonary exudates that progressed to pulmonary hemorrhage. Gastrointestinal bleeding and worsening of DIC persisted. Despite aggressive cardiopulmonary and hemodynamic support, including nitric oxide inhalation, vasopressors (dopamine, norepinephrine, and dobutamine), and milrinone, the clinical condition continued to decline.

Microbiological workup, including throat, stool, and blood cultures, as well as cerebrospinal fluid analysis, were negative for bacteria and viruses. Metagenomic next-generation sequencing (mNGS) failed to identify any infectious agent. In view of intrapartum maternal fever, maternal infectious studies beyond routine intrapartum care were not obtained at delivery as clinical efforts prioritized neonatal stabilization. Collectively, sterile blood/CSF cultures, as well as negative plasma mNGS, did not support an infectious trigger for secondary HLH. Targeted TORCH serologies/PCR were not obtained prior to death because the patient remained hemodynamically unstable with escalating transfusion requirements, and the limited interval before death did not permit additional venipuncture beyond clinically essential draws. Consultations were coordinated by the NICU team; the extracorporeal membrane oxygenation (ECMO) service participated in escalation discussions, and the family declined ECMO. The infant died on day 5 of life.

### 2.6 Genetic analysis

The study was conducted in accordance with the ethical principles and guidelines outlined by the Ethics Review Committee of Hangzhou First People’s Hospital under approval number “2025ZN220-1.” These principles are consistent with the “International Ethical Guidelines for Health-Related Research Involving Humans,” the Declaration of Helsinki. Ante-mortem sequencing was not initiated because the clinical window was too short for our center’s ∼4-week turnaround of standard clinical WES and ultra-rapid trio WES/WGS was not available; severe coagulopathy with ongoing transfusions also made additional sampling impractical. With parental consent, post-mortem whole-exome sequencing established the molecular diagnosis (biallelic PRF1), enabling targeted family counselling and cascade testing.

- c.1131C>A (p.Cys377Ter), inherited from the father
- c.65delC (p.Pro22Argfs*29), inherited from the mother

**FIGURE 1:**
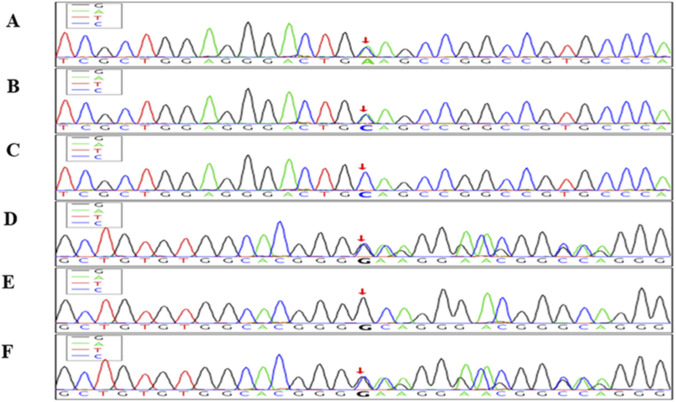
Sanger sequencing chromatograms showing compound heterozygous mutations in the PRF1 gene in the index case and parents. The top three panels **(A–C)** show sequencing results for the PRF1 c.1131C>A (p.Cys377Ter) nonsense mutation. **(A)** Index case: heterozygous C>A transition at nucleotide position c.1131 (red arrow). **(B)** Father: heterozygous for the same C>A mutation. **(C)** Mother: wild-type sequence (CC). The bottom three panels **(D–F)** display the PRF1 c.65delC (p.Pro22Argfs*29) frameshift mutation. **(D)** Index case: heterozygous deletion of cytosine at position c.65 (red arrow). **(E)** Father: wild-type sequence. **(F)** Mother: heterozygous deletion of cytosine. These chromatograms confirm that the index case carries compound heterozygous mutations in PRF1, consistent with autosomal recessive familial hemophagocytic lymphohistiocytosis type 2 (FHL2). Red arrows indicate the exact mutation sites.

Both variants were predicted to disrupt protein function and were consistent with the diagnosis of familial hemophagocytic lymphohistiocytosis type 2 (FHL2). These findings were confirmed by trio analysis, which demonstrated an autosomal recessive inheritance pattern, with each parent being a heterozygous carrier. Detailed variant data and inheritance patterns are presented in Table 2, and a mutation schematic is illustrated in Figure 1.

**TABLE 2:** Genetic variants identified in the PRF1 gene in the index case and parents.

Specimen	Gene	Genomic position (hg19)	cDNA change	Reference base	Variant	Zygosity
Index case	PRF1	chr10:72358346	c.1131C>A	C	CA	Heterozygous
Father	PRF1	chr10:72358346	c.1131C>A	C	CA	Heterozygous
Mother	PRF1	chr10:72358346	c.1131C>A	C	CC	Wild-type
Index case	PRF1	chr10:72360593	c.65delC	C	delC	Heterozygous
Father	PRF1	chr10:72360593	c.65delC	C	CC	Wild-type
Mother	PRF1	chr10:72360593	c.65delC	C	delC	Heterozygous

Chromosomal microarray (CMA) and SNP array are optimized to detect aneuploidies, pathogenic copy-number variants above platform resolution, and, on SNP platforms, large regions of homozygosity or uniparental disomy. However, these array-based assays do not interrogate single-nucleotide variants (SNVs) or small insertions/deletions. They therefore would not identify the nonsense and frameshift PRF1 variants detected by sequencing in this case. Balanced rearrangements and low-level mosaicism may also escape detection. A negative prenatal microarray is thus compatible with a subsequent monogenic diagnosis by sequencing.

The family received counselling regarding the autosomal-recessive inheritance of PRF1-related disease, a 25% recurrence risk, and options for targeted prenatal diagnosis of the two familial PRF1 variants (c.1131C>A, c.65delC) via chorionic villus sampling (11–13 weeks) or amniocentesis (≥15 weeks); once samples are received, focused assays directed at these variants can typically be reported within a few business days. Preimplantation genetic testing (PGT-M) was discussed as a preconception option. We emphasized that cfDNA/NIPT and chromosomal microarray do not exclude single-gene PRF1 variants and are therefore insufficient to rule out FHL2. In our setting, ultra-rapid trio WES/WGS returns in ∼48–72 h, rapid targeted NGS panels in ∼3–7 days, and standard clinical WES in ∼4 weeks.

## 3 Discussion

HLH is a hyperinflammatory syndrome in which familial (primary) forms arise from biallelic variants in cytotoxic-pathway genes (e.g., *PRF1*, *UNC13D*, *STX11*, *STXBP2*), and in neonates the presentation may closely mimic sepsis (Zhang et al., 2024).

According to these guidelines, HLH can be diagnosed if at least 5/8 of the criteria are met or if there are molecular findings consistent with fHLH (i.e., biallelic loss-making mutations in the fHLH gene) (Henter et al., 2007).

We assessed the proband against HLH-2004 guidance rather than reproducing the full criteria here. Although sIL-2R and NK-cell function were unobtainable because of coagulopathy and a very short clinical window, the combination of fever, bicytopenia, hyperferritinemia, hypofibrinogenemia, rapidly escalating organ dysfunction, and postmortem confirmation of biallelic PRF1 loss collectively supported a diagnosis of familial HLH (FHL2) in the neonatal period.

In our patient, the HLH cytopenia criterion was partially met: severe thrombocytopenia (30 × 10^9^/L) was present, whereas hemoglobin (132 g/L) and ANC (3.28 × 10^9^/L) did not meet threshold values; thus 1/3 lineages met the criterion on recorded labs. Supportive criteria were fulfilled by hypertriglyceridemia (3.78 mmol/L), hypofibrinogenemia (0.77 g/L), and hyperferritinemia (>40,000 μg/L). In neonates, serial rather than single CRP measurements improve interpretive value, and a normal early CRP does not exclude severe inflammatory disease (Boscarino et al., 2023; Briggs-Steinberg and Roth, 2023; Fleiss et al., 2023; Kilpatrick et al., 2025).

The diagnosis of fHLH is challenging because its manifestations are similar to those of other inflammatory, infectious, and malignant diseases (Bode et al., 2015). However, it is crucial to distinguish between fHLH and sHLH, as fHLH can only be cured by allogeneic hematopoietic stem cell transplantation (HSCT). In addition, it is necessary to distinguish fHLH from a variety of similar diseases, including congenital immune system deficiencies (such as SH2D1A and XIAP-related lymphoproliferative disorders), combined immune deficiencies (RAG1 and IL27RA), hereditary inflammasome deficiencies (NLRC4 and NLRP3, classically presenting as neonatal-onset multisystem inflammatory disease [NOMID/CINCA]) (Goldbach-Mansky et al., 2006; Wm et al., 2007), and metabolic disorders (SLC7A7, GBA, and LIPA), as well as malignant processes such as congenital leukemia (Brown et al., 2019; Brown, 2021). In sepsis-negative neonates with early multisystem inflammation, the differential should therefore include monogenic autoinflammatory syndromes (NLRP3/NOMID (Goldbach-Mansky et al., 2006; Wm et al., 2007), NLRC4 gain-of-function (Canna et al., 2014; Romberg et al., 2014)), primary immunodeficiencies (e.g., SH2D1A/XIAP, RAG1/IL27RA), inborn errors of metabolism, and congenital leukemia (Brown et al., 2019; Brown, 2021), alongside familial HLH. Fortunately, the advancement and application of genomic technologies (such as next-generation sequencing NGS, and microarray analysis) have made it possible to distinguish between these diseases. Notably, microarrays interrogate copy–number–level changes and regions of homozygosity, whereas sequencing is required to detect pathogenic single-nucleotide variants and small indels in genes such as *PRF1*. Where available, ultra-rapid trio WES/WGS (≤48–72 h) may guide management in suspected familial HLH; however, availability, consent logistics, sample-volume limitations, and competing resuscitative priorities can preclude ante-mortem sequencing in fulminant neonatal courses. In our fulminant course, key supportive studies were obtained (ferritin, triglycerides, fibrinogen), whereas sIL-2R and NK-cell activity were unavailable; molecular confirmation of biallelic *PRF1* variants provided definitive diagnostic resolution. We evaluated potential secondary HLH, particularly infection and inborn errors of metabolism. Negative blood/CSF cultures and negative mNGS argued against an infectious precipitant; TORCH testing was not obtained before death, and TMS/GC-MS was negative; in this context, biallelic *PRF1* variants provided a definitive explanation for familial HLH (FHL2), and a secondary trigger was not identified. Antenatal HLH has been reported rarely; in this family, there were no data to implicate an *in-utero* presentation.

Taken together, these diagnostic constraints explain the sepsis-like presentation and the reliance on sequencing in fulminant neonatal FHL2, a pattern mirrored in prior case reports and summaries (Zhang et al., 2024). Prior reports describe FHL2 in neonates and young infants who initially mimic sepsis and then deteriorate rapidly with cytopenias, coagulopathy, hepatosplenomegaly, and multiorgan failure. These include a fatal neonatal FHL2 with compound *PRF1* variants and absent perforin expression, and an early-infant FHL2 that presented as late-onset neonatal sepsis before genetic confirmation. These observations align with our patient’s fulminant course and underscore that perforin deficiency can clinically resemble severe neonatal sepsis (Kim et al., 2014; Ramitha et al., 2024).

A Chinese neonatal FHL2 case with literature review identified 75 Chinese FHL2 patients, highlighting recurrent *PRF1* hotspots, c.1349C>T (p.Thr450Met) (≈24.8%) and c.65delC (≈11.6%). In a separate pediatric series from a national center, *PRF1* accounted for ∼34% of genetically defined primary HLH. Recurrent alleles and potential regional/founder effects may contribute to the higher reported case counts, although ascertainment and center effects likely play a role. Our patient’s genotype (c.1131C>A; c.65delC) fits within this spectrum that includes c.65delC among Chinese FHL2 reports (Ma et al., 2020; Bi et al., 2021).

Our proband carried compound heterozygous truncating *PRF1* variants, c.1131C>A (p.Cys377Ter) and c.65delC (p.Pro22Argfs*29), confirmed in trans. Truncating *PRF1* alleles are loss-of-function and align with neonatal-/early-infant-onset FHL2; notably, c.65delC has been reported among recurrent alleles in Chinese cohorts, and neonatal, sepsis-like courses have been described in FHL2 (Kim et al., 2014; Ma et al., 2020; Bi et al., 2021; Zhang et al., 2024).

*PRF1* encodes perforin, a pore-forming effector essential for cytotoxic lymphocyte function; loss-of-function variants abrogate perforin expression or activity, via nonsense-mediated decay or truncation of critical domains, producing the immunopathology characteristic of FHL2 (Rudd-Schmidt et al., 2019; Zhang et al., 2024). In this context, a nonsense allele (p.Cys377Ter) and an early frameshift (p.Pro22Argfs*29) provide a coherent molecular explanation for the fulminant neonatal presentation in our case. The dominant negative effect of the mutant protein may occur, but this is rare (Spessott et al., 2015). Therefore, fHLH is caused by mutations in perforin and its upstream specific genes; however, determining the association between specific mutations and phenotypes may be challenging.

The NGS panel is the preferred detection solution for fHLH, as it can simultaneously analyze multiple HLH-related genes and improve the diagnostic rate. These panels usually have high detection sensitivity for point mutations and nucleotide insertions/deletions, but have limited detection capabilities for non-coding regions, structural variations, and large fragment deletions/duplications. For example, the *UNC13D* gene in FHL3 may be inactivated due to deep intron mutations and complex insertions. These variations cannot be detected through an NGS panel (Meeths et al., 2011), and special detection and/or whole-genome sequencing designed for each structural variation is required. Finally, conventional detection of unconfirmed cases can involve the whole exome sequencing (WES) or whole genome sequencing (WGS) (Stavropoulos et al., 2016). WES covers almost all genomic coding regions, whereas WGS can also detect non-coding regions and structural variations, making it currently the most sensitive detection method. Accordingly, while microarray remains complementary for aneuploidy and larger CNVs, it will not capture the majority of monogenic PRF1 variants that are readily identified by sequencing-based assays.

The conventional initial treatment for fHLH is based on the HLH-94 clinical trial, combined with etoposide and dexamethasone. If there is evidence of central nervous system (CNS) involvement that is progressive after 2 weeks of systemic therapy and/or persistent abnormal cerebrospinal fluid, intrathecal methotrexate (±hydrocortisone) is administered at weeks 3, 4, 5, and 6 per HLH-94 (Trottestam et al., 2011). Subsequently, dexamethasone, etoposide, and cyclosporine (CSA) were used as bridging and maintenance treatments to continuously control the disease until HCT (Trottestam et al., 2011). In addition, for patients with CNS involvement, intrathecal corticosteroids and methotrexate are often used in combination (according to the HLH-2004 clinical trial).

In familial HLH, etoposide-based HLH-94/2004 chemo-immunotherapy is used as a bridge to curative HSCT (Henter et al., 2007; Trottestam et al., 2011; Ehl et al., 2018), and outcomes are best when disease is controlled at transplant (Trottestam et al., 2011; Lucchini et al., 2018; Yanagisawa et al., 2019; Pegoraro et al., 2024). In our neonate, the fulminant 5-day course with profound coagulopathy precluded completion of HLH-2004 testing, initiation of induction therapy, or consideration of HSCT.

## 4 Conclusion

We report a fulminant neonatal FHL2 case associated with biallelic PRF1 variants that initially mimicked sepsis. The trajectory limited HLH-2004 labs and ante-mortem sequencing; diagnosis was secured post-mortem by WES. This experience highlights the limitations of prenatal microarrays for monogenic diseases, the need to prioritize rapid sequencing when FHL is suspected, and the importance of family counseling with cascade testing. Early recognition and prompt sequencing are pivotal, with HSCT as the curative pathway once the disease is controlled.

## Data Availability

The data supporting the findings of this case report are available from the corresponding author upon reasonable request.
